# The ‘Erlenmeter’: a low-cost, open-source turbidimeter for no-sampling phenotyping of microorganism growth

**DOI:** 10.7717/peerj.17659

**Published:** 2024-07-09

**Authors:** João Serôdio, Alexandra Bastos, Silja Frankenbach, Jörg C. Frommlet, Ana Cristina Esteves, Henrique Queiroga

**Affiliations:** CESAM–Centre for Environmental and Marine Studies and Department of Biology, University of Aveiro, Aveiro, Portugal

**Keywords:** Microalgae, Cultures, Erlenmeyer, Bacteria, Growth curves, Science education, Yeasts

## Abstract

This work presents a low-cost, open-source turbidimeter, the ‘Erlenmeter’, designed to monitor the growth of microorganisms in batch cultures. It is easy to build, based exclusively on inexpensive off-the-shelf electronic components and 3D-printed parts. The Erlenmeter allows measuring the optical density of cultures on standard Erlenmeyer flasks without the need to open the flasks to collect aliquots, ensuring speed, minimal use of consumables, and elimination of the risk of contamination. These features make it particularly well-suited not just for routine research assays but also for experimental teaching. Here we illustrate the use of the Erlenmeter turbidimeter to record the growth of the microalga *Phaeodactylum tricornutum*, of the bacterium *Escherichia coli*, and of the yeast *Saccharomyces cerevisiae*, model organisms that are widely used in research and teaching. The Erlenmeter allows a detailed characterization of the growth curves of all organisms, confirming its usefulness for studying microbial populations dynamics both for research purposes and in classroom settings.

## Introduction

The study of the growth dynamics of microorganisms such as bacteria, microalgae or yeasts, plays an important role in both scientific research and science education. It is of interest for a wide range of research fields, including biochemistry, cell physiology, microbiology, genetics, ecology, toxicology, and evolution (Henley, 2019). The growth rate of microbial populations is a particularly important quantitative trait for microbial ecology and evolution, that has been used for characterizing the comparative inclusive fitness of different populations (Wood, Everroad & Wingard, 2005), for evaluating the responses to selection in experimental evolution studies (Hall et al., 2014), and for investigating the competition for limited resources (Ram et al., 2019). It is key for population genetics models, and central in phenomics (Wood, Everroad & Wingard, 2005; Hall et al., 2014).

The quantitative study of microbial population growth is also of interest for science education (Forget et al., 2010). It provides a ground for manipulative experimentation with live organisms, including planning and execution of experiments, as well as the collection, analysis, and interpretation of biological data. It also provides an opportunity to demonstrate important theoretical biological principles regarding population dynamics, resource limitation, or competition, while providing experimental data amenable to introductory biostatistics techniques. All this in experiments that typically give consistent results, are relatively simple and inexpensive, and are easy to carry out in a classroom context using commonly available model microorganisms.

The growth of microbial populations is generally studied by quantifying the variation of biomass over time in batch cultures, by measuring cell concentration or, alternatively, proxies that are easier to measure such as absorbance, turbidity or, for photosynthetic organisms, *in vivo* chlorophyll fluorescence (Wood, Everroad & Wingard, 2005; Hall et al., 2014). By far the most used method is to measure optical density (OD) at specific wavelengths, using a turbidimeter, a spectrophotometer or a plate reader. Batch cultures have long been used for this purpose as they offer numerous advantages, including being inexpensive, requiring low volume of growth media, and allowing easy and versatile manipulations (Wood, Everroad & Wingard, 2005; Henley, 2019).

The spectrophotometric determination of OD often involves the frequent sterile collection of aliquots from the batch culture vessels, increasing the risk of culture contamination, requires large amounts of laboratory consumables (pipette tips, spectrophotometer cuvettes), and has the undesired effect of reducing the culture volume over time. This may not only limit the temporal resolution of the experiments but may also change the culture conditions during the growth period. A common form of avoiding the need to open the cultures vessels is to use special Erlenmeyer flasks that have a protuberant sidearm, or the so-called nephelometric flasks (Scherr, 1952), which can be directly inserted in specific commercial turbidimeters or spectrophotometers. However, besides having the disadvantage of having to use low culture volumes, because of the need to be tilted during OD determination, this approach does not eliminate the need to use a commercial instrument to carry out the OD measurements.

Here we present a low-cost, open-source, and easy-to-build turbidimeter, designed around the Erlenmeyer flask, the type of vessel typically used for batch cultures of microorganisms, and thus named ‘Erlenmeter’. Unlike commercially available options and other open-source turbidimeters, the presented model is easy to construct, based on inexpensive off-the-shelf parts and 3D-printed parts, and does not require advanced engineer training, making it particularly suitable for experimental teaching. An appealing feature of the Erlenmeter is that it allows measuring the OD of cultures directly on standard Erlenmeyer flasks without the need to open them to collect aliquots, thus avoiding all the above-mentioned disadvantages associated with culture sampling. These features are particularly useful in the context of experimental teaching, where access to commercial turbidimeters can be difficult and the sterile manipulation of cultures by students may be especially challenging.

Besides providing a detailed description of the turbidimeter, its parts, and how to build it, this article illustrates its use by quantifying the growth kinetics of three microorganisms grown in batch culture, the microalga *Phaeodactylum tricornutum*, the bacterium *Escherichia coli* ATCC 25922, and the yeast *Saccharomyces cerevisiae* S288C, all easy to obtain and often used in research and teaching. The application of the turbidimeter in a classroom setting was illustrated with a study of the growth response of phytoplankton microalgae to limiting factors, demonstrating its potential for diverse pedagogically interesting exercises.

## Materials & Methods

### Design and fabrication

**Figure 1:**
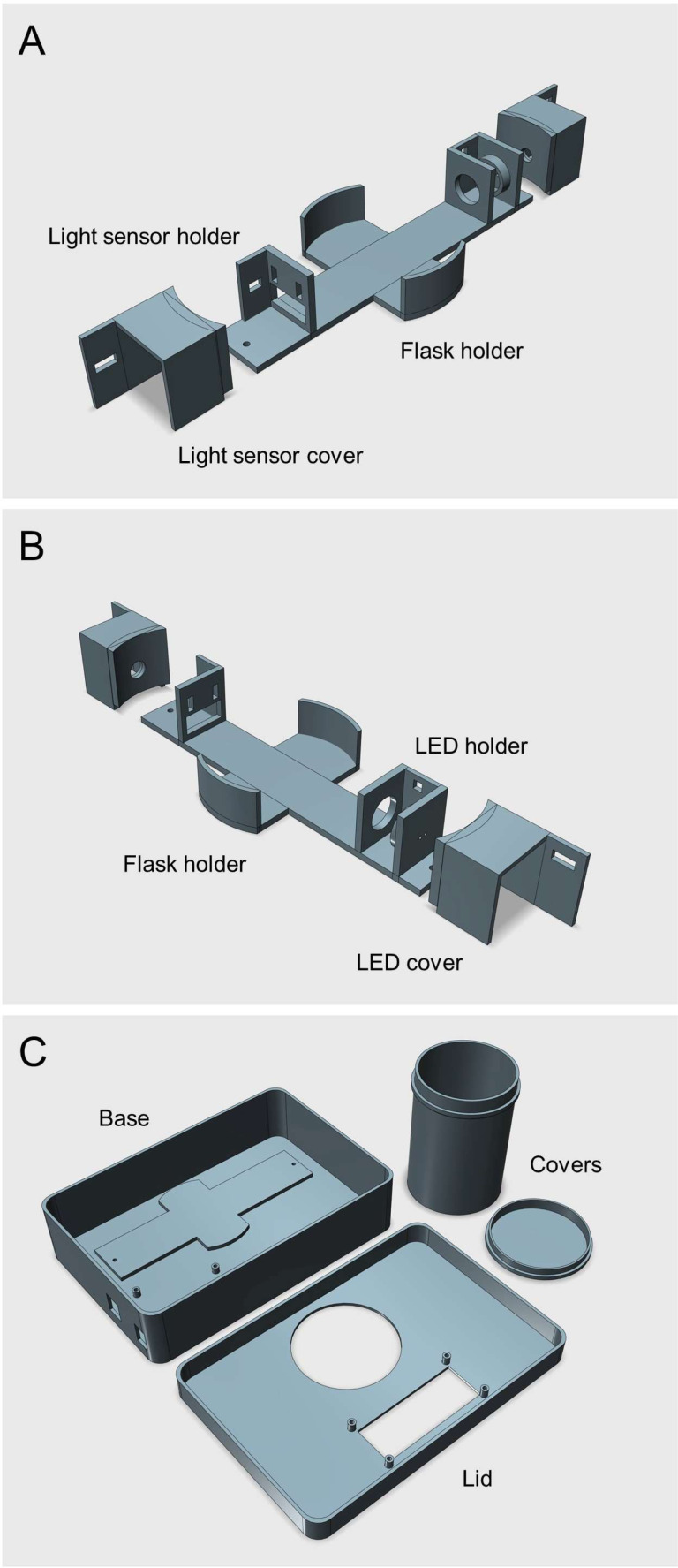
3D rendered model of the Erlenmeter turbidimeter. (A–B) Main frame of the Erlenmeter turbidimeter, showing the positioning of the LED and of the light sensor, in opposed positions relative to the Erlenmeyer flask, placed in the center. Both the LED and the light sensor are placed inside covers to reduce stray light from reaching the sensor. The LED and light sensor covers are fixed to the main frame using M2 screws and bolts, allowing adjustments of the distance between the two to accommodate Erlenmeyer flasks of different diameters. (C) Housing where the main frame is placed, eliminating ambient light reaching the light sensor. All parts were 3D printed in black matte PLA to minimize light reflections inside the housing and stray light reaching the light sensor (STL files provided as Supplemental Information).

The Erlenmeter turbidimeter is composed of an LED (generic Ultra Bright 10 mm LED) and a light sensor (Adafruit TSL 2591; http://www.adafruit.com/product/1980), placed in opposing positions around the Erlenmeyer flask, so that the light beam emitted by the LED crosses the center of the flask, at its maximum width, and is detected by the light sensor (Fig. 1).

A microcontroller (Arduino UNO R3; http://www.arduino.cc) is used to control the LED, register the signals received by the light sensor, compute the OD, and display its value on an LCD display (generic 16x2 I2C LCD module). Two types of LEDs were used: one blue (peaking at 466 nm) and one orange (peaking at 598 nm), for measuring OD in microalgae and in bacteria and yeast cultures, respectively (Fig. S1). The reason for using a blue LED for measuring OD in microalgae was to match the highest absorption peak of the main photosynthetic pigment, chlorophyll *a*. Being the growth medium of the microalgae colorless (as opposed to the ones used for bacteria and yeast), the use of blue light maximizes the sensitivity of the instrument.

**Figure 2:**
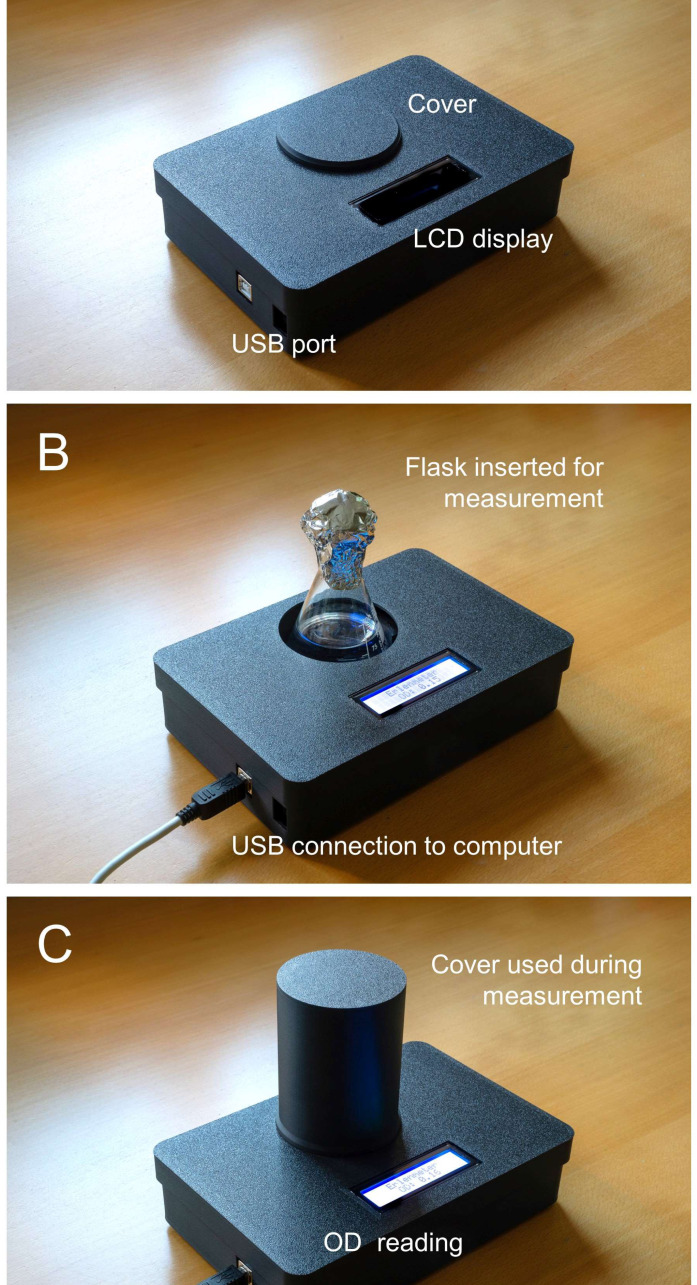
Photographs of the Erlenmeter turbidimeter. (A) Assembled 3D-printed housing, USB cable for connecting the microcontroller to a computer, and dust cover for when stored or inactive; (B) the mode of insertion of Erlenmeyer flask for OD measurement; (C) use of flask cover for OD reading.

To direct the light beam into the flask and to limit stray light from reaching the sensor, both the LED and the light sensor are placed inside casings with a small circular opening. To further minimize stray light from reaching the light sensor, the whole system is placed inside a closed housing, with one main opening for inserting the flask and another for powering and connecting the microcontroller to a computer *via* USB (Fig. 2A, B). During operation, the flask is covered by an opaque lid to block ambient light (Fig. 2C).

All parts were 3D printed (Bambu Lab P1P; Bambu Lab GmbH, Frankfurt am Main, Germany) using black matte PLA filament (PLA Matte, Bambu Lab), to minimize light reflections. STL files are available as supplemental material. The parts can be printed using any commercial 3D-printer and any type of filament (preferably black).

**Figure 3:**
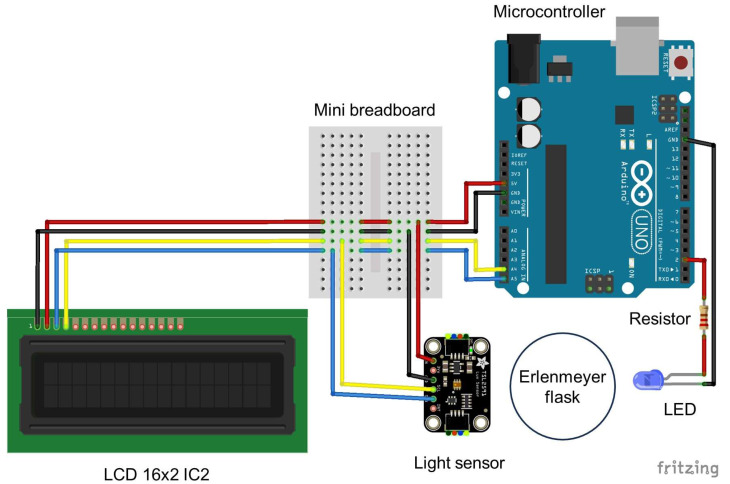
Wiring diagram for the Erlenmeter turbidimeter. Indication of the position of the Erlenmeyer flask in between the LED and the light sensor and the remaining components: an Arduino microcontroller, a breadboard and an LCD display. The schematic was created using Fritzing 0.9.3 (http://fritzing.org).

The light sensor was fixed in position using two M2 screws and bolts. The LED was fixed in position using a 10 mm LED mounting clip. A list of components is given in Table 1 and a wiring diagram is presented in Fig. 3. The Arduino IDE code used to control the turbidimeter is provided as supplemental material. The spectra of the LEDs were measured using a spectroradiometer (SpectraPen Mini; Photon Systems Instruments, Drásov, Czech Republic).

**Table 1:** List of components. In case of generic parts, main specifications are given. Approximate prices. Total price <60 €.

Component	Model	Cost (€)
Microcontroller	Arduino UNO R3 (http://www.arduino.cc)	30
Light sensor	Adafruit TSL 2961 (http://www.adafruit.com/product/439)	12
LCD	16 ×2 I2C LCD module	10
Orange LED	Ultra Bright Red 10 mm LED (598 nm)	<1
Blue LED	Ultra Bright Blue 10 mm LED (480 nm)	<1
Mini breadboard		<2
220 Ω resistor		<1
10 mm LED holder		<1

### Microorganisms and experiments

To illustrate the variety of potential applications of the turbidimeter, several experiments were carried out, using three microorganisms, the photoautotrophic marine diatom *Phaeodactylum tricornutum* Bohlin 1898 (culture collection of the Department of Biology of the University of Aveiro), the bacterium *Escherichia coli* (Migula) Castellani and Chalmers ATCC 25922, and the yeast *Saccharomices cerevisiae* S288C. Three experiments were carried out on batch cultures under controlled conditions, using *P. tricornutum* (Experiment 1), *E. coli* (Experiment 2) and *S. cerevisiae* (Experiment 3). These experiments were designed to demonstrate the ability to characterize in detail the growth dynamics of different types of microorganisms with contrasting growth rates. A fourth experiment was conducted to illustrate the use of the Erlenmeter turbidimeter for monitoring growth and to determine dilutions for semicontinuous culturing, using *P. tricornutum* (Experiment 4).

A final experiment illustrates the application of the Erlenmeter turbidimeter in a classroom environment, using a factorial design to test the effects of two abiotic factors, light intensity (2 levels) and nutrient concentration (4 levels), on the growth of *P. tricornutum* (Experiment 5). Cultures were grown on a shelf of a north-facing window of a classroom, where maximum irradiance reached ca. 200 *μ* mol photons m^−2^ s^−1^ and temperature varied between 17 and 22 °C. The two light levels were achieved by having one set of flasks directly exposed to ambient light coming from the window (L2) while another set of flasks was shaded by double dark grey mosquito nets that reduced incident light by ca. 50% (L1). The nutrient concentration of the growth medium was modified by diluting the original f/2 medium to obtain four levels: 0.0 (seawater; N0), 0.05 (N1), 0.1 (N2) and 0.5 (N3).

*Phaeodactylum tricornutum* was grown in 100 mL Erlenmeyer flasks in f/2 medium (Guillard & Ryther, 1962) with double Si content, prepared from natural seawater, in a growth chamber (MLR-352-PE Climate Chamber; Panasonic, Osaka, Japan) under 80 µmol photons m^−2^ s^−1^ and a 12h:12 h photoperiod, at 18 °C. During experiment 1, OD was measured at the end of the light period and at the end of the dark period.

*Escherichia. coli* ATCC 25922 was grown according to Tuttle, Trahan & Son (2021) with slight modifications. Briefly, a pre-inoculum was made from one colony of a fresh culture of *E. coli* ATCC 25922 grown in Tryptic Soy Agar (TSA) in 10 mL of Tryptic Soy Broth (TSB) and incubated overnight (16–18h) at 37 °C, at 180 rpm. Afterwards, a 1:2500 dilution was made in TSB and *E. coli* was grown in 75 mL of TSB medium, in 100 mL Erlenmeyer flasks at 37 °C, under constant agitation (180 rpm).

*Saccharomyces cerevisiae* S288C, was grown according to Corbacho et al. (2011) with slight modifications. Briefly, a pre-inoculum was made from one colony of a fresh culture of *S. cerevisiae* grown in Yeast Peptone Dextrose Agar (YPDA) in 10 mL of Yeast Peptone Dextrose Broth (YPDB) and incubated overnight (19h) at 30 °C, at 180 rpm. Afterwards, a 1:2500 dilution was made in YPDB and *S. cerevisiae* was grown in 75 mL of TSB medium, in 100 mL Erlenmeyer flasks at 30 °C, under constant agitation (180 rpm).

All measures were taken in triplicates. To verify the existence of a linear relationship between OD and cell concentration, at the end of Experiments 1 and 3, the cultures were sequentially diluted to 10 equidistant concentrations and OD was measured for each concentration.

### OD measurements

Before starting each experiment, a blank (flask containing only growth medium) was used to zero the turbidimeter, by determining a reference value so that, when assigned to the variable ‘ReadZero’ in the microcontroller Arduino IDE code, result in OD reading of 0. This was done separately for each LED, blue or orange, using the respective growth medium (blue LED: f/2 medium; orange LED: TSB/YPDB).

In the case of *P. tricornutum* cultures, OD was measured daily, close to the end of the photoperiod. For *E. coli* and *S. cerevisiae*, measurements were made at 30 and 60 min intervals, respectively, until reaching the stationary phase. Flasks were removed one by one from the growth chamber (*P. tricornutum*) or shaker (*E. coli* and *S. cerevisiae*), measured, and each flask was immediately returned, limiting the time that cultures were removed from their regular growth conditions to less than 30 s. In the case of *P. tricornutum*, flasks were manually stirred before each measurement. A blank measurement was made before each set of measurements, and the resulting value was later subtracted from the OD of the sample measured on the same occasion. Furthermore, the reading made at the start of each growth curve was subtracted from the subsequent readings. To minimize variation in OD measurements between replicates due to differences in flask shape, all Erlenmeyer flasks were of the same model (100 mL narrow neck Simax; Kavalierglass, Sázava, Czech Republic). The position of the flasks was carefully checked to avoid having the lettering or other marks in the glass in the path of the measuring light, interfering with the OD reading.

### Comparison with a standard spectrophotometer

To further ascertain the validity of the Erlenmeter turbidimeter, OD measurements obtained with it were compared to OD measurements obtained with a conventional spectrophotometer, using standard 1 cm path cuvettes (Genesys 6; Thermo Spectronic, Cambridge, MA, USA). Measurements were made using the two LEDs, the blue one on *P. tricornutum* suspensions and the orange on *E. coli* and *S. cerevisiae* suspension. Full grown cultures were sequentially diluted to 10 decreasing concentrations, and, for each concentration, the OD was measured using the Erlenmeter and the spectrophotometer. This approach was preferred to the paired measurement of ODs during the growth of the cultures because that would imply the removal of aliquots for the spectrophotometer leading to a cumulative reduction of the culture volume, which would impact the readings in the Erlenmeter.

### Parameterization of culture growth

Growth curves were analyzed following a kinetic approach in which a function explicitly describing the variation of the population size over time was fitted to the entire curve (Ghenu, Marrec & Bank, 2024). The growth of the *P. tricornutum*, *E. coli* and *S. cerevisiae* cultures was described by fitting a logistic model and estimating the parameters *μ*, *K* and *L*: *μ* is the intrinsic growth rate (also often denoted as *r*), measuring the highest number of divisions per capita and per unit of time theoretically possible (h^−1^ or d^−1^); *K* is the carrying capacity, the maximum population size reached at the stationary phase, when nutrients became depleted; *L* is a parameter quantifying the duration of the lag phase in the start of the exponential phase of growth (h or d). The following formulation of the logistic model was used:

(1) \begin{eqnarray*}N \left( t \right) = \frac{K}{1+ \frac{K-N(0)}{N(0)} {e}^{-\mu (t-L)}} +N(0)\end{eqnarray*}

where *N*(0) and *N*(t) are the cell concentrations (estimated by OD) at the beginning and at time *t*, respectively. This is a simplified version of the model recently proposed by Reding-Roman et al. (2017). The model was fitted to experimental data and the model parameters were estimated by iteratively minimizing a least-squares function using MS Excel Solver.

### Statistical analysis

Differences among mean values of parameters *μ* and *K* were tested by applying two-way ANOVA. The existence of linear relationships between OD and relative concentration was tested by linear regression analysis.

## Results

**Figure 4:**
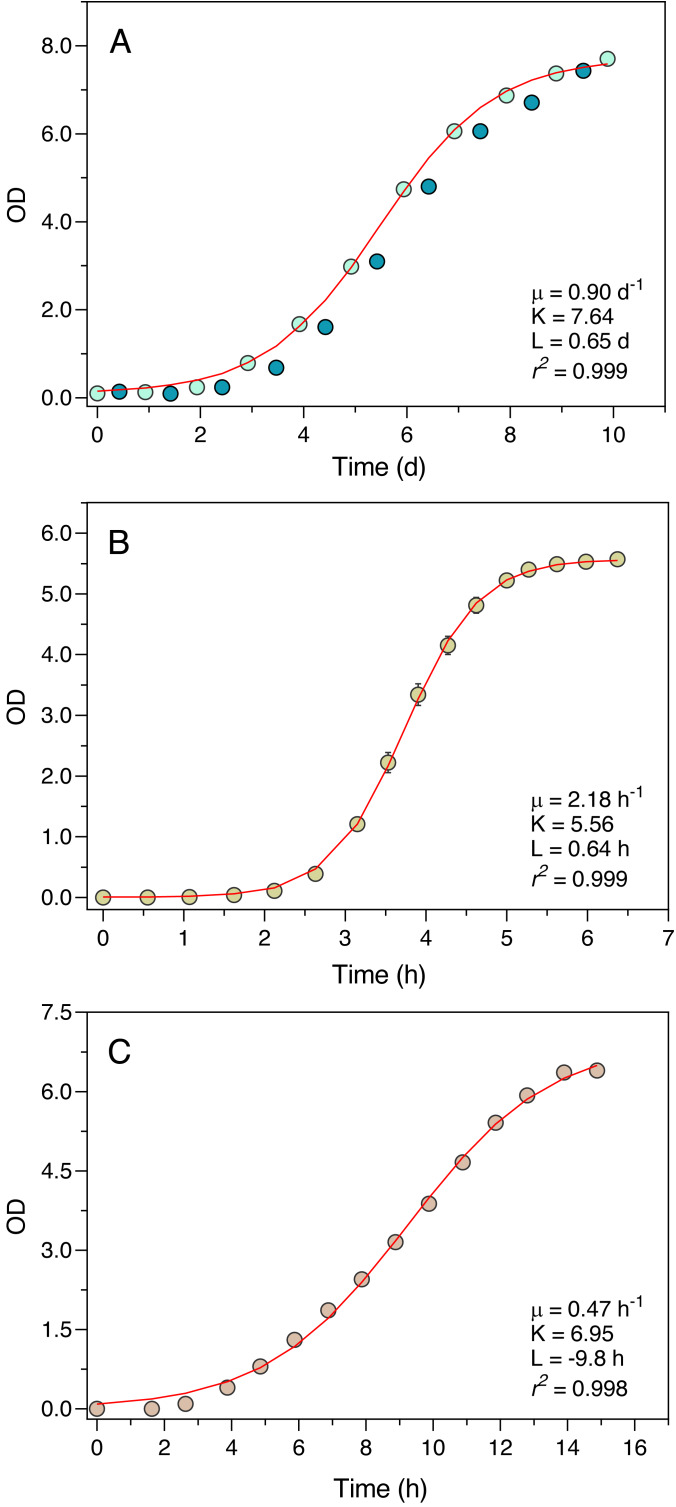
Growth curves of *P. tricornutum* (A), E. coli ATCC 25922 (B) and S. cerevisiae (C) as derived from OD measurements recorded using the Erlenmeter turbidimeter. In the case of *P. tricornutum* (A), the daily variation of OD measured at the end of the light periods (light blue data points) and at the end of the night periods (dark blue data points) is depicted. The red lines represent the fit of the logistic model of Eq. (1) (for *P. tricornutum*, fitted to the measurements made at the end of the light period) resulting in the indicated parameter estimates. Mean values of three replicates. Error bars represent one standard deviation.

The Erlenmeter turbidimeter allowed for the detailed characterization of the growth curve of all organisms tested (Fig. 4). For *P. tricornutum* (Fig. 4A), the values of OD recorded at the end of the day periods (light blue data points) were very closely described by the logistic model of Eq. (1), as confirmed by the excellent fit between model predictions and observations (*r*^2^ = 0.999). The intrinsic growth rate reached *μ* = 0.90 d^−1^, leading to a maximum OD (carrying capacity) of *K* = 7.64. Measurements made at the end of the night periods (dark blue data points) revealed an almost complete interruption of growth during the dark periods, in some cases even resulting in a reduction of the OD values.

Also, in the case of *E. coli* and *S. cerevisiae* (Experiments 2 and 3) the turbidimeter was able to produce a detailed and well-defined growth curve, with little variation between replicates (c.v. < 1% in all cases) (Figs. 4B, 4C). The logistic model of Eq. (1) provided an excellent fit to the data (*r*^2^ > 0.998 in both cases), resulting in the estimation of intrinsic growth rates of *μ* = 2.18 h^−1^ and *μ* = 0.47 h^−1^, and carrying capacities of *K* = 5.56 and *K* = 6.95, for *E. coli* and *S. cerevisiae*, respectively.

**Figure 5:**
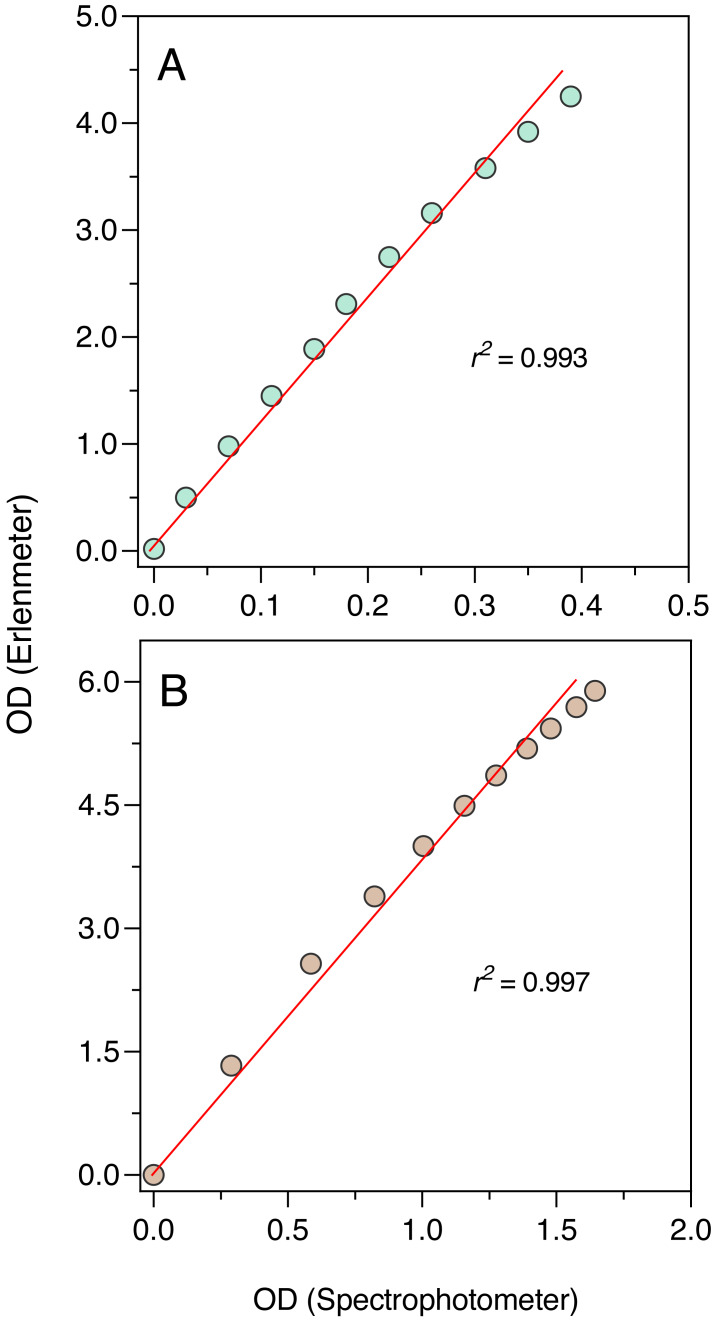
Growth curve of *E. coli* ATCC 25922 as derived from OD measurements recorded using the Erlenmeter turbidimeter. Hourly variation of OD (A) and of ln(OD) (B). The red lines represent the fit of the logistic model of Eq. (1), resulting in the indicated parameter estimates (A) and the slope of the first seven data points, corresponding to the linear part of the curve (B). Mean values of three replicates. Error bars represent one standard deviation.

The comparison between the OD measurements from the Erlenmeter turbidimeter and the spectrophotometer revealed highly significant linear relationships both for the blue and orange measuring light (*P* < 0.001; Fig. 5). In both cases, a slight curvature under the higher OD values could be observed, likely because of self-shading caused by the longer light path of the Erlenmeter. This was however too weak to compromise the use of the turbidimeter to characterize the growth of cultures.

**Figure 6:**
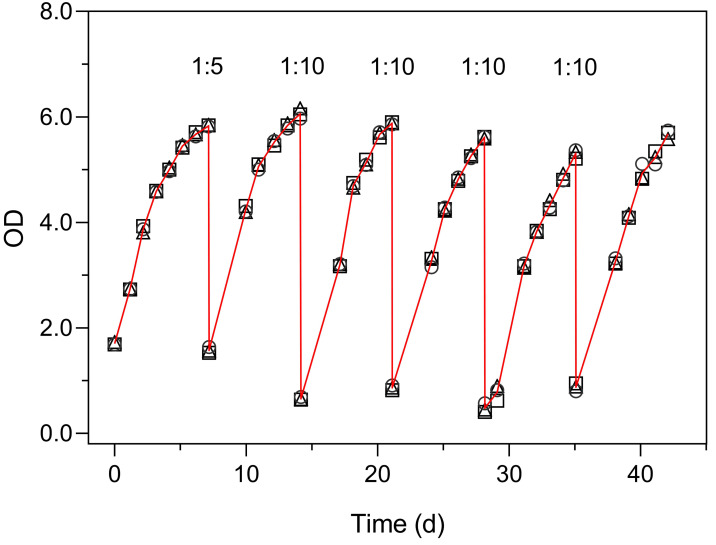
Use of the Erlenmeter turbidimeter for the maintenance of semicontinuous cultures, through the monitoring of OD and the periodic dilution of cultures of *P. tricornutum*. Each symbol represents a separate culture vessel. Numbers indicate the dilution applied at each step.

Figure 6 illustrates the use of the turbidimeter for the maintenance of semicontinuous cultures (Experiment 4). This experiment enabled monitoring the growth of the culture and to detect the onset of reduced growth rates, without the need to open the flasks and thus avoiding the reduction in the culture volume over prolonged periods (over 40 days). These results also demonstrate the very low inter-vessel variability (c. v. < 3% on average).

**Figure 7:**
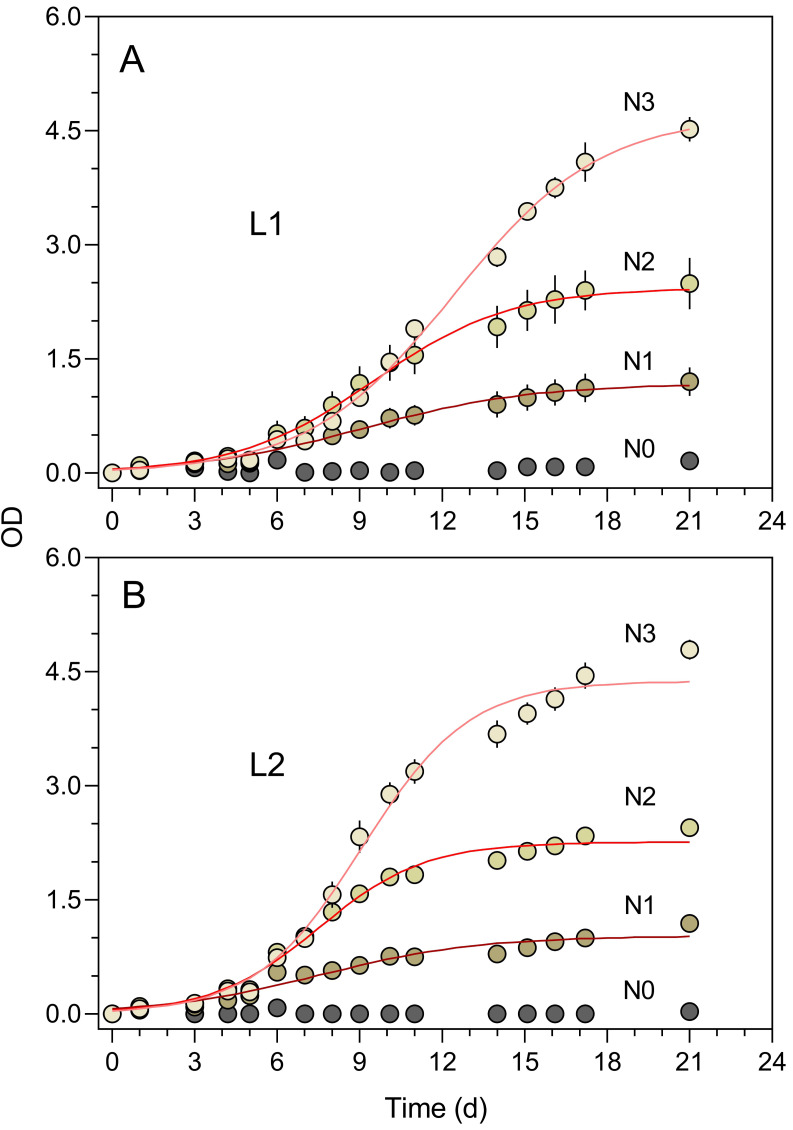
Growth curves of *P. tricornutum* batch cultures grown under different light and nutrient levels. Two light levels (L1, 50% ambient light (A); L2, 100% ambient light (B)) and four levels of growth medium nutrient concentration (from N0 to N3) were applied. The red lines represent the fit of the logistic model of Eq. (1) to the various light and nutrient conditions (N1 to N3). Mean values of five replicates. Error bars represent one standard deviation.

The turbidimeter allowed for detecting clear differences between *P. tricornutum* growth under different light conditions and nutrient levels carried out in the classroom context (Experiment 4, Fig. 7). Marked effects of growth light were recorded, with the cultures exposed to 50% ambient light (L1, Fig. 7A) showing a later and slower increase in OD than those grown under 100% light conditions (L2, Fig. 7B). In both light conditions, clear effects of the nutrient concentration were also registered, with virtually no growth being detected for N0 and a marked increase in OD levels with increasing nutrient levels (N1 to N3).

The growth curves of Fig. 6 present an aspect that can be expected when monitoring the relatively slow growth of microalgae in a classroom setting: the gaps in the time series of observations due to the interruption of the daily measurements on weekends. The lack of flask stirring during these days is likely the cause for the observed decrease in growth during these periods. Despite these interruptions, the fit of Eq. (1) was very good, and the resulting data set was fully usable for further analysis and interpretation.

**Figure 8:**
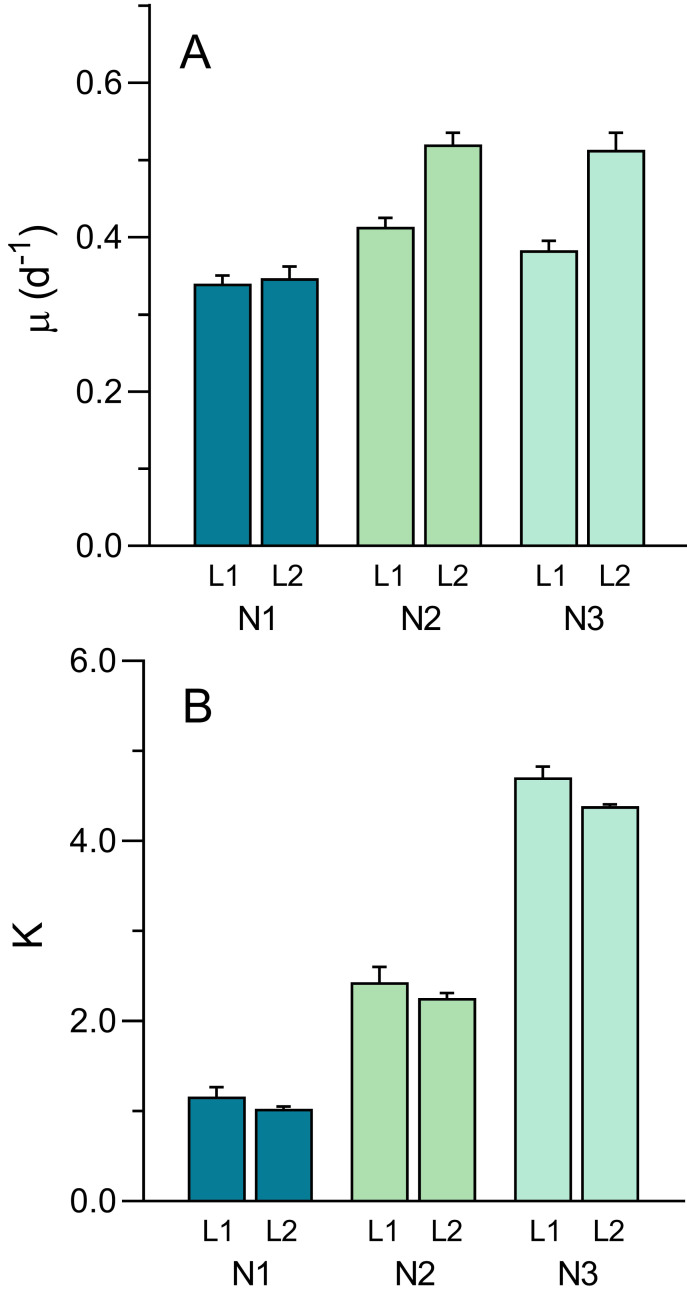
Variation of the growth curve parameters *μ* (A) and K (B) with light (L1, L2) and nutrient levels (N1–N3) of *P. tricornutum* batch cultures. Parameters estimated obtained from fitting Eq. (1) to the growth curves in Fig. 6. Mean values of five replicates. Error bars represent one standard deviation.

The well-defined patterns of growth allowed for the fitting the logistic model (Eq. 1) and the estimation of parameters *μ* and *K* (Fig. 8). The intrinsic growth rate *μ* was significantly affected both by light intensity and nutrient level (two-way ANOVA; *F*_1,12_ = 45.61, *p* < 0.001 and *F*_1,12_ = 40.44, *p* < 0.001, respectively). *μ* increased with light intensity (0.379 ± 0.036 d^−1^ to 0.46 ± 0.09 d^−1^, from L1 to L2) and increased with nutrient level, although mostly between N1 (0.34 ± 0.02 d^−1^) and N2 or N3 (0.47 ± 0.06 d^−1^ and 0.45 ± 0.08 d^−1^, respectively) (Fig. 8A). A significant interaction was found between the two factors (*F*_2,12_ = 9.83, *p* = 0.003), indicating that the same increase in nutrient level caused a higher increase in *μ*_max_ under high light than under low light, suggesting light limitation of culture growth.

While *μ* was more affected by light than nutrients, the opposite happened with the carrying capacity, *K* (Fig. 8B). Effects of light intensity were relatively small, although still significant (*F*_1,12_ = 6.50, *p* = 0.026), resulting from a decrease in *K* with light level, from 2.77 ± 1.47 (L1) to 2.56 ± 1.57 (L2). This was possibly caused by the fact that, due to the slower growth under L1 conditions, the growth curve measurements were interrupted before reaching the maximum OD values thus resulting in the underestimation of the real value of *K*. In contrast, nutrient levels had a marked effect on *K*, that increased significantly from N1 to N3, regardless of the light conditions applied (*F*_2,12_ = 614.3. *p* < 0.001), doubling from each nutrient level to the next (1.10 ± 0.14, 2.36 ± 0.22, and 4.55 ± 0.22 for N1, N2 and N3 conditions, respectively). No significant interaction was found between the two factors, indicating that the increase in *K* with increasing nutrients followed essentially the same trend under the two light conditions.

## Discussion

Though simple in design, the Erlenmeter turbidimeter is a valuable tool for phenotyping the growth of microbial populations in batch culture. It is easy to use, facilitating fast measurements on multiple samples with minimal effort, on standard Erlenmeyer flasks, and easy and inexpensive to build, making it particularly well-suited for teaching. Main advantages of this turbidimeter include its no-sampling feature, ensuring speed, minimal use of consumables, and elimination of the risk of contamination. This allows for an increase in the time resolution of the measurements, as well as the ability to handle a large number of samples, treatments, and replications. The turbidimeter also stands out for its exclusive use of off-the-shelf electronic components and 3D-printed parts, resulting in a low-cost, do-it-yourself (DIY) solution.

The possibility to measure OD without the need to open the culture vessels is especially valuable in a classroom setting, as it reduces the risk of accidental contamination. The non-destructive nature of the measurements further allows for repetitive monitoring without losing sample volume, a feature invaluable when conducting repeated measurements multiple times. In a classroom context, the Erlenmeter enables the acquisition of comprehensive datasets on microbial growth dynamics and their dependence on environmental variables, providing an opportunity for introducing students to the use of statistical analysis methods such as non-linear model fitting and ANOVA.

### Comparison with alternative models

Other open-source and inexpensive turbidity models have been published but they are either not easy to build, requiring custom-made circuitry (Sperandio et al., 2022) or require sampling the culture and use of cuvettes for carrying out the OD measurements (Kelley et al., 2014). Furthermore, these models were designed for measuring the concentration of chemical compounds and were not tested for monitoring microorganism growth.

Several commercial systems that allow measurement of OD in closed Erlenmeyer flasks are also available (*e.g.*, http://www.scientificbio.com/cgq, http://www.essi.tech/shake-flaskod, https://bionexus.net/od-monitor-turbidostat-bl.html; http://www.presens.de/products/detail/sfr-vario) but these are, by nature, not low-cost alternatives, and are based on proprietary hardware and software.

### Growth curves and parameters

The main desired use of a turbidimeter like the Erlenmeter is the phenotyping for quantitative traits related to microbial population dynamics, particularly the ability to reliably measure growth curves and estimate parameters such as growth rates or carrying capacities. The results presented here show that the Erlenmeter turbidimeter can generate well-defined and detailed growth curves enabling the precise determination of growth parameters, as demonstrated by the consistent low inter-sample variability and the excellent fitting of theoretical models.

We illustrated how the turbidimeter can be used to estimate growth parameters, considering the kinetic approach based on the fitting of theoretical models to the whole growth curve.

The fit of the logistic model, the most commonly used kinetic model (Ghenu, Marrec & Bank, 2024), to the growth curves generated by the Erlenmeter turbidimeter resulted in very good fits in all cases, allowing the concurrent estimation of the curve parameters *μ*, *K* and *L*. In regard to *μ*, the estimates obtained in this study for *P. tricornutum* (0.35 –0.90 d^−1^) align with the values available in the literature using commercial instruments (0.35–1.43 d^−1^; (Rech et al., 2005; Ragni & D’Alcalà, 2007; Wu, Gao & Riebesell, 2010; Wagner et al., 2016; Schoefs et al., 2017). The same is true for *E. coli* and *S. cerevisiae*. For *E. coli*, the estimate of this study (2.2 h^−1^) is well within the values reported in the literature for the same growth conditions (doubling times of ∼20 min, corresponding to growth rates of 2.08 h^−1^ (Tuttle, Trahan & Son, 2021). In the case of *S. cerevisiae*, the estimated growth rate matches the range of published values for this yeast under comparable conditions (0.03–0.5 h^−1^; Win, Impoolsup & Noomhorn, 1996; Boender et al., 2009).

The kinetic approach allows to further estimate the parameters *K* and *L*. The carrying capacity *K*, although largely dependent and specific on experimental factors independent of the biology of the organisms (vessel capacity, nutrient load, *etc*.) and thus not generalizable to different environments or conditions, is still of interest to characterize the maximum reachable population size at the stationary phase when all the usable resources are depleted. This is especially true within the same experiment, when *K* can be directly compared among treatments to assess the effect of experimental conditions on the biology of the organisms. It must be noted, however, that the values of K derived from using a turbidimeter are directly based on OD readings and its full biological meaning can only be achieved after converting OD to cell concentration or other measure of biomass.

### Limitations

Despite its many advantages, the described turbidimeter suffers from potential issues and limitations. The main assumption of using a turbidimeter to quantify the growth of a microbial culture is the existence of a linear relationship between OD and cell concentration (or other direct measure of biomass) along the range of values attained during culture growth (Wood, Everroad & Wingard, 2005; Hall et al., 2014).

The excellent correlation observed between the OD measurements made with the Erlenmeter and a standard spectrophotometer, both for the blue and orange measuring lights, ensures that the former can be confidently used to characterize the growth of microalgae, bacteria and yeasts. However, the deviation from linearity observed for higher concentrations indicates that while the fitting of the logistic model to the whole growth curve can be used to confidently estimate parameter *μ*, some underestimation of the value of *K* may occur. The reasons for this lack of linearity for the higher range of OD values are likely related to the large light scattering caused by the relatively long path of the light traversing the Erlenmeyer flask, which is aggravated under high cell concentrations. In can be noted that the longer light path of the Erlenmeter turbidimeter might in fact represent an advantage: by increasing the chances that cells are detected by the measuring light beam, this design may enable a better characterization of the culture growth in its early stages, when OD is still very low.

The establishment of the relationship between the OD measurements obtained by the two instruments also allows to convert the Erlenmeter readings, necessarily higher values due to the longer light path, to standardized (1-cm light path) OD values.

### Further applications

The Erlenmeter turbidimeter can be applied to organisms and growth conditions other than those used in this article, such as other yeasts, bacteria or microalgae, as well as cyanobacteria. In the case of microalgae and cyanobacteria, the color of the LED should be chosen carefully as there is a large diversity in the coloration, and thus spectral absorption characteristics, between the various algae groups. For example, blue LEDs should be avoided for measuring the OD of cyanobacteria as the wavelengths in this spectral region are poorly absorbed by the piments of these cells, causing a loss of sensitivity. In this case, red LEDs should be preferred, as red radiation is well absorbed by chlorophyll *a*.

For each different type of microorganism, growth medium, and LED color, it is recommended that the light sensor settings are adjusted to guarantee optimal detection of light across the whole range of levels expected during the growth of the culture. The sensor used in the Erlenmeter turbidimeter can be adjusted in terms of sensitivity and integration time, which allows the user to find the right balance between detecting low cell concentrations at the initial stages of growth and avoiding limiting out the sensor at the stationary phase.

Despite its designation and chosen configuration, the turbidimeter can be easily adapted for using other types of culture flasks. Erlenmeyer flasks are advantageous in many situations due to their capacity to be swirled vigorously without spilling, but other cultures vessels may be preferred depending on the particular needs of each study. The Erlenmeter can be readily adapted simply by designing and 3D-printing the main frame parts holding the flask between the LED and the light sensor. It is recommended to test different culture volumes as, depending on the flask shape, small volumes may interfere with the OD reading.

While this article was centered on the validation and on the uses of the Erlenmeter in experimental research and science teaching, the building of the turbidimeter can be in itself an interesting pedagogic project. We provide detailed assembly and manufacturing instructions and a comprehensive list of affordable and accessible components that make it easy to build the Erlenmeter turbidimeter by students in the classroom context.

## Conclusions

The Erlenmeter allows the detailed characterization of the population dynamics of microorganisms grown in batch culture. Both microalgae, yeasts and bacteria can be easily phenotyped in terms of growth kinetics, enabling to quantify the growth curve parameters *μ* (intrinsic growth rate) and K (carrying capacity). Being exclusively based on off-the-shelf and 3D-printed parts, the Erlenmeter represents an inexpensive and easy to build alternative to commercial instruments, being particularly suited not only for routine research assays but also for use in a classroom setting.

## Supplemental Information

10.7717/peerj.17659/supp-1 Supplemental Information 1 Emission spectra of the LEDs used in this work Blue line is the emission spectrum of the blue LED used for growing the microalga *P. tricornutum* and the orange line is the emission spectrum of the orange LED used to grow the bacteria *E. coli* and the yeast *S. cerevisiae*. Numbers represent the wavelength of maximum intensity.

10.7717/peerj.17659/supp-2 Supplemental Information 2 STL and STEP files for 3D printing STL and STEP files for 3D printing the main frame of the Erlenmeter turbidimeter, including holders and covers for the LED and the light sensor, and the housing. The opening allows the use of flasks with diameters up to 68 mm, however it can be adjusted for other dimensions in the file.

10.7717/peerj.17659/supp-3 Supplemental Information 3 Arduino IDE code used for programming the Arduino microcontroller

10.7717/peerj.17659/supp-4 Supplemental Information 4 Raw Data Raw data used for Figs. 5–9.

## Additional Information and Declarations

### Data Availability

The following information was supplied regarding data availability: The raw data is available in the Supplemental File.
