# Supplementary figures and images for: The ‘Erlenmeter’: a low-cost, open-source turbidimeter for no-sampling phenotyping of microorganism growth
Source: PeerJ. 2024 Jul 9;12:e17659. doi: 10.7717/peerj.17659 (PMC11243968; doi:10.7717/peerj.17659)

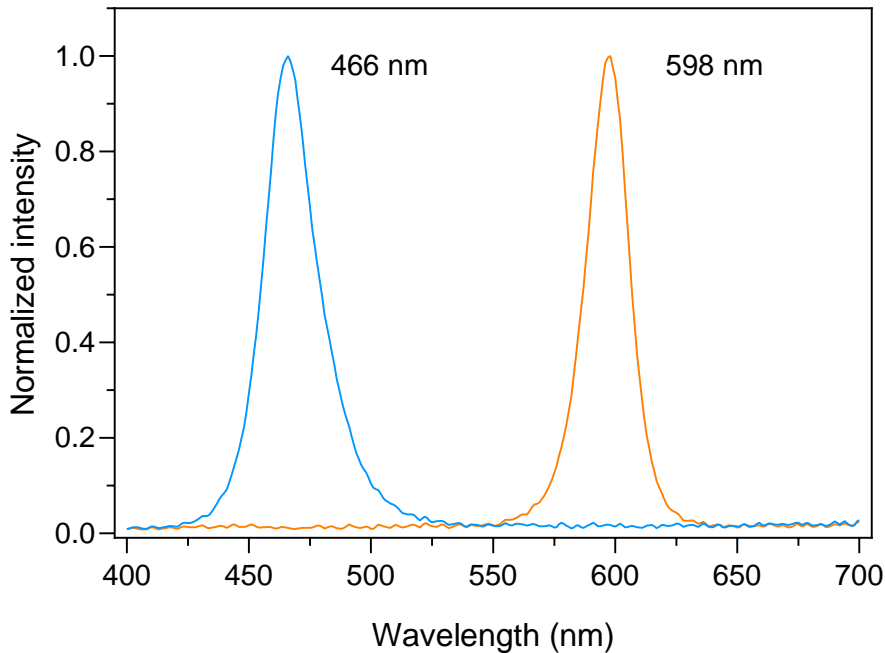

Supplement: Supplemental Information 1 — Blue line is the emission spectrum of the blue LED used for growing the microalga P. tricornutum and the orange line is the emission spectrum of the orange LED used to grow the bacteria E. coli and the yeast S. cerevisiae. Numbers represent the wavelength of maximum intensity. [file peerj-12-17659-s001.pdf]
